# Editorial: Elicitors, secret agents at the service of the plant kingdom

**DOI:** 10.3389/fpls.2022.1060483

**Published:** 2022-10-18

**Authors:** Essaid Ait Barka, Rachid Lahlali, Brigitte Mauch-Mani

**Affiliations:** ^1^ Unité de Recherche Résistance Induite et Bio-Protection des Plantes-EA 4707-USC INRAE1488, Reims Champagne-Ardenne University, Reims, France; ^2^ Department of Plant Protection, Phytopathology Unit, Ecole Nationale d’Agriculture de Meknès, Meknès, Morocco; ^3^ Institute of Biology, University of Neuchâtel, Neuchâtel, Switzerland

**Keywords:** effectors, pathogen-associated molecular pattern, pattern recognition receptors, plant defense, resistance

Due to their sessile lifestyle plants had to acquire skills enabling them to defend themselves against a wide range of various stresses. At the very beginning of a successful defense is the capability to perceive a given stressor that will lead to the mounting and activation of defense responses (Jones and Dangl, 2006). The self/nonself perception in plants in defence is tightly linked to the presence of the plants’ own innate immune system (Sanabria et al., 2010). In this system, a first layer of immunity is based on the perception of pathogen-associated molecular patterns (PAMPs) or microbe-associated molecular patterns (MAMPs) and generally referred to as PAMP-triggered immunity (PTI) (Couto and Zipfel, 2016).

An additional layer of defense is based on the ability of plants to being primed (Mauch-Mani et al., 2017). In priming, elicitors derived from pathogens, beneficial microorganisms, or in the form of various synthetic and natural compounds sensitize the plant cells to react more rapidly or/and accurately to a future challenging infection. Most of such so-called exogenous elicitors of plant defense responses are nonspecific and fluctuate widely in their chemical nature including proteins, glycoproteins, oligosaccharides, and lipids (Abdul Malik et al., 2020).

Since elicitors help to protect plants from diseases by triggering their immune system, they also represent encouraging alternatives to pesticides and are in line with today’s requirement for more sustainability in agriculture. Therefore, the current topic presents various suggestions for promising candidates that could be integrated into a future low-pesticide approaches to plant protection.

Until recently, the function of *VirE3* of Agrobacterium in the plant hormone signal transduction pathway for tumorigenesis was not known. In their contribution, Li et al. show that Agrobacterium VirE3 can interact with Arabidopsis JAZ8 in cells, leading to a repression of the transcription activity of *VirE3* by JAZ8. The authors suggest that *VirE3* and *JAZ8* may antagonistically modulate the SA/JA mediated plant defense signaling response during Agrobacterium infection.

In the search for new elicitors, the sea might constitute a good source. In this context, de Borba et al. report for that foliar application of ulvan, a characterized water-soluble polysaccharide from the green seaweed *Ulva fasciata*, induces resistance in wheat against *Zymoseptoria tritici*, the causing agent of *Septoria tritici* blotch (STB), that leads to high economic losses. Ulvan treatment interestingly did not cause substantial changes in the wheat metabolome suggesting only low metabolic costs for this induced resistance.

Not only microbial pathogens but also nematodes are able to cause damage to plants. Singh et al. show in their contribution that ascorbate oxidation in rice, known to induce resistance (Singh et al., 2020), enhances the phenylpropanoid-based response to nematode infection and leads to a tolerance phenotype in treated rice plants.

In their contribution, Nakamura et al. showed that effectors can play different roles in host and non-host plants. The bacterial strains *Acidovorax avenae* N1141 and K1 produce an effector called protein rice HR cell death inducing-factor (RHIF). This novel effector RHIFs performs in both establishing infection in host plants and inducing ETI in non-host plants.

SOBIR1 (Suppressor Of BIR1) is a receptor-like kinase (Gao et al., 2009) that associates with a plethora of pattern-recognition receptors (PRRs) of the receptor-like protein (RLP) type at the plasma membrane. Based on bimolecular fluorescence complementation and affinity purification assays, Li et al. established that *Nb*RLP1 associates with *Nb*SOBIR1. The presented data support a model where *Nb*RLP1 acts as a positive regulator for plant PTI through its binding with *Nb*SOBIR1.

Pathogenic microbes release a body of effector proteins which manipulate host immunity to successfully colonize the plants. In this way, the *Valsa mali* effector VmEP1 is an essential virulence factor for the establishment of apple Valsa canker (Li et al.). Wang et al. present evidence for a mechanism by which a *V. mali* effector protein targets the host pathogenesis-related protein 10 to attenuate the host resistance.

The citrus disease Huanglongbing (HLB) caused by *Candidatus Liberibacter asiaticus* seriously threatens citrus production (Duan et al., 2009). Microbial Fermentation Application (MFA), an elicitor program, is formulated with a bacterial fermentation medium, yeast cell wall extract and a Cu component has been shown to reduce infection rates in other crops (Twamley et al.). Lally et al. show that MFA can stabilise HLB infection and increase the expression of important defence pathways in citrus under field conditions, including multiple PR genes, lignin formation genes, ROS-related genes, hormone synthases, and hormone regulators, providing therefore important evidence where MFA may play an pivotal role as a plant elicitor in the battle against HLB in citrus but also in other agronomically important crops.

Arabinogalactan proteins (AGPs) are cell wall resident glycosylated proteins found in land plants, and which are known to play a role in several plant biological functions. Přerovská et al. have isolated an arabinogalactan protein-like (AGP-like) enriched fraction from *Ulva lactuca* and assessed its ability to protect oilseed rape (*B. napus*) cotyledons against *Leptosphaeria maculans*, and to activate the host immune responses. Preventive application of the Ulva AGP-like enriched fraction on oilseed rape, followed by cotyledon inoculation with *L. maculans*, significantly reduces the progress of infection. The authors conclude that *U. lactuca* AGP-like glycoproteins exhibit promising elicitor activity and that plant eliciting properties of sea lettuce extract, might result not only from an ulvan-originated eliciting proprieties, but also be AGP-like originated.

Damage-associated molecular patterns (DAMPs) are danger signals released from the damaged host tissue or present on the surface of stressed cells (Bhat and Ryu, 2016). In their contribution, Kim et al. investigate self-extracellular RNA (eRNA) as a danger signal in plants. Their field experiments in pepper against viral and bacterial pathogens demonstrate, that self-eRNA can successfully trigger plant systemic immunity without any growth penalty, suggesting the potential of eRNA as a novel disease management agent against a wild range of pathogenic microbes.

## Author contributions

All authors listed have made a substantial, direct, and intellectual contribution to the work and approved it for publication.

## Conflict of interest

The authors declare that the research was conducted in the absence of any commercial or financial relationships that could be construed as a potential conflict of interest.

## Publisher’s note

All claims expressed in this article are solely those of the authors and do not necessarily represent those of their affiliated organizations, or those of the publisher, the editors and the reviewers. Any product that may be evaluated in this article, or claim that may be made by its manufacturer, is not guaranteed or endorsed by the publisher.
